# Identification of Chalcone Synthase Genes and Their Responses to Salt and Cold Stress in *Poncirus trifoliata*

**DOI:** 10.3390/plants14193003

**Published:** 2025-09-28

**Authors:** Lijuan Jiang, Yu Sheng, Chengyang Song, Teng Liu, Shuangyu Sheng, Xiaoyong Xu

**Affiliations:** College of Horticulture and Landscape Architecture, Yangzhou University, Yangzhou 225009, China; 008318@yzu.edu.cn (L.J.); mz120231501@stu.yzu.edu.cn (Y.S.); 221806212@stu.yzu.edu.cn (C.S.); 191803315@stu.yzu.edu.cn (T.L.); mz120231500@stu.yzu.edu.cn (S.S.)

**Keywords:** bioinformatics analysis, *CHS* gene family, *Poncirus trifoliata*, stress response

## Abstract

Chalcone Synthase (CHS) plays a vital role in flavonoid synthesis, influencing plant growth, development, and responses to both biotic and abiotic stress. In this study, 11 *CHS* genes were identified in *Poncirus trifoliata* using bioinformatics methods, with their distribution across five chromosomes and unassigned contigs. Each gene contains 2–3 exons and 3–8 conserved motifs. In silico prediction suggested that the PtrCHS proteins are localized in the cytoplasm. PtrCHS9 and PtrCHS11 share identical protein tertiary structures. Phylogenetic analysis classified the *CHS* family members into four subgroups. Synteny analysis revealed one set of collinear gene pairs within *Poncirus trifoliata*. Between *Poncirus trifoliata* and *Arabidopsis thaliana*, two sets of collinear gene pairs were identified, while one such set was found between *Poncirus trifoliata* and *Oryza sativa*. Promoter element analysis showed the presence of various hormone response and stress response elements within *PtrCHS* promoters. RNA-Seq data demonstrated tissue-specific expression patterns of *PtrCHSs*. RT-qPCR results indicated that all *CHS* genes, except *PtrCHS11*, respond to salt stress with dynamic, member-specific patterns. Additionally, four *PtrCHSs* (*PtrCHS3*, *PtrCHS5*, *PtrCHS7*, and *PtrCHS10*) were significantly upregulated in response to cold treatment. Notably, *PtrCHS7* and *PtrCHS10* maintained high expression levels at both 6 and 12 h, implying they may be key players in cold stress response in *Poncirus trifoliata*. Clones of *PtrCHS7* and *PtrCHS10* were obtained, and overexpression vectors were constructed in preparation for gene transformation. Overall, this study provides a solid foundation for future research into the functions of the *PtrCHSs*.

## 1. Introduction

In recent years, with the intensification of global climate change and the expansion of farmland salinization, plants have been increasingly exposed to severe abiotic stress [1]. Environmental stress affects the physiological metabolism of plants through various mechanisms, all of which trigger the activation of the plant’s complex internal defense network [2]. Among these mechanisms, the flavonoid metabolic pathway, a key branch of secondary metabolism, plays a vital role in maintaining physiological functions, responding to biotic and abiotic stress, and regulating growth and development [3]. Its metabolites, such as flavonols, not only scavenge ROS but also stabilize the cell membrane structure, thereby enhancing stress resistance in plants. Anthocyanins accumulate in plant tissues exposed to cold stress, offering protection against cold-induced photoinhibition and oxidative stress [4].

Chalcone synthase (CHS) is the first key rate-limiting enzyme in the flavonoid biosynthetic pathway in plants, catalyzing the formation of chalcone from malonyl-CoA and coumaroyl-CoA [5]. Encoded by a multigene family, CHS has been extensively identified and characterized across plant species owing to advances in high-throughput sequencing and bioinformatics. For instance, monocotyledonous plants such as rice and maizecontain 27 and 14 *CHS* members, respectively [6,7], while dicotyledonous plants including soybean, eggplant, chrysanthemum, and common bean possess 14, 7, 16, and 14 *CHS* members, respectively [8,9,10,11]. Furthermore, *CHS* expression is regulated by diverse environmental and hormonal cues. Under salt stress, transgenic tobacco overexpressing *NtCHS1* exhibited elevated rutin accumulation, which correlated with enhanced ROS scavenging capacity, reduced hydrogen peroxide and superoxide anion levels, and improved survival rates [12]. Similarly, the *AcMYB176*-*AcCHS5* regulatory module has been shown to enhance salt tolerance by orchestrating flavonoid biosynthesis and ROS scavenging [13]. The responsiveness of *CHS* to salinity is further evidenced in maize, where the expression level of *ZmCHS25* was upregulated 9.3-fold after 24 h of salt stress [14]. Beyond salinity, *CHS* is also involved in cold stress adaptation. For example, the transcript level of the *CHS* gene was significantly increased in Columbia-0 (Col-0) seedlings after a 24 h treatment at 4 °C [15], a finding consistent with observations in walnut, where the expression of *JrCHS4-CHS6* was significantly increased following cold stress [16]. Similarly, *MdCHS* promotes the accumulation of apple peel anthocyanins in response to UV-B radiation and low temperatures [17]. The involvement of *CHS* extends to other abiotic stresses as well. In tobacco, *NtCHS6* expression is induced by light and drought [18], and mulberry *MaCHS* improves tolerance to drought, and heat stress in transgenic tobacco [19]. The expression of grape *VvCHS* is inhibited by high nighttime temperatures, thereby affecting anthocyanins biosynthesis [20]. Moreover, *CHS* expression can be modulated by hormonal signals, such as methyl jasmonate (MeJA) upregulates the expression levels of *ZbCHS71*, *ZbCHS43*, and *ZbCHS26* in *Zanthoxylum bungeanum*, directly promoting the synthesis and accumulation of flavonoid metabolites [21]. Additionally, transcriptional regulators such as *CmMYB9a* positively regulate *CmCHS* to promote pigmentation in chrysanthemum [22]. These findings underscore the crucial roles of *CHS* in plant development and stress adaptation.

Citrus (*Citrus* spp.), belonging to the family Rutaceae, is widely cultivated around the world. It is rich in Vitamin C and serves as a major source of health-promoting compounds. China accounts for approximately 33.3% of global citrus production [23]. Citrus is highly sensitive to temperature; low temperatures can limit plant growth and development, impair photosynthesis, and reduce fruit quality [24,25,26]. At the same time, the issue of soil salinization is becoming increasingly serious. Most species within the citrus genus have a shallow root system and are particularly vulnerable to salt stress. High-salinity conditions can result in ion toxicity, osmotic imbalance, and oxidative stress, which may subsequently lead to leaf yellowing, growth stagnation, or even plant death [27]. Both low temperature and high salinity significantly impact the yield and quality of citrus, representing major environmental challenges to the long-term and stable development of the citrus industry.

In recent years, the development of resistant and high-quality fruit tree germplasm through genetic engineering has gradually become an efficient and feasible approach. The discovery and identification of resistance-related genes in citrus are essential prerequisites for achieving germplasm improvement via genetic engineering. The main types of citruses include orange, mandarin, pomelo, kumquat, lemon, and *Poncirus trifoliata*. Among them, *Poncirus trifoliata* is the most important rootstock type for citrus, and has been extensively studied for its ability to significantly enhance the cold resistance of grafting scion variety and its moderate salt tolerance [28]. Therefore, the use of *Poncirus trifoliata* as a material for identifying cold resistance genes holds great theoretical and practical significance for citrus cold resistance breeding.

At present, significant progress has been made in research on citrus salt tolerance and cold resistance genes, with numerous related genes having been identified and studied, including *bZIPs*, *bHLH18*, *ICE1*, *ERF109*, *ZAT12*, *PIF8*, and *CBF* [29,30,31,32,33,34,35]. For example, the overexpression of *PtbZIP49* from *Poncirus trifoliata* has been shown to enhance the salt tolerance of transgenic *Arabidopsis* [30]. Similarly, overexpression of *PRpnp* has significantly improved the salt tolerance of transgenic *Citrus aurantifolia* [36]. In terms of cold resistance, *PtrERF9*, a cold-responsive ethylene-induced transcription factor in *Poncirus trifoliata*, facilitates ROS scavenging and regulates downstream genes to enhance cold tolerance [37]. *PtrERF108* positively modulates raffinose synthesis via *PtrRafS* to confer cold resistance [38], and *PtrBAM1*, a member of the CBF regulatory network, plays an important role in cold tolerance by regulating the levels of soluble sugars [28]. *Poncirus trifoliata* has developed specific cold tolerance through both C-repeat/DREB binding factor (CBF)-dependent and CBF-independent cold signaling pathways [39]. Nevertheless, salt and cold tolerance in citrus are complex quantitative traits governed by multiple genes, and additional related genes remain to be discovered and investigated.

Currently, no systematic identification or analysis of the *CHS* gene family members in *Poncirus trifoliata* has been reported, and their response patterns to salt stress and cold stress remain unclear. Therefore, in this study, bioinformatics approaches were used to identify all *CHS* gene members in *Poncirus trifoliata*, and to analyze their physicochemical properties, gene structures, protein structures, promoter cis-acting elements, and tissue-specific expression profiles. In addition, their gene expression patterns under salt stress and cold stress were investigated. The goal is to identify key *CHS* genes involved in salt and cold stress responses, thereby providing a theoretical foundation for studies on the salt tolerance and cold resistance function of *CHS* genes in *Poncirus trifoliata*, and offering a reference for resistance breeding improvement in citrus.

## 2. Results

### 2.1. Identification and Physicochemical Property Prediction of CHS Family Members

**Figure 1:**
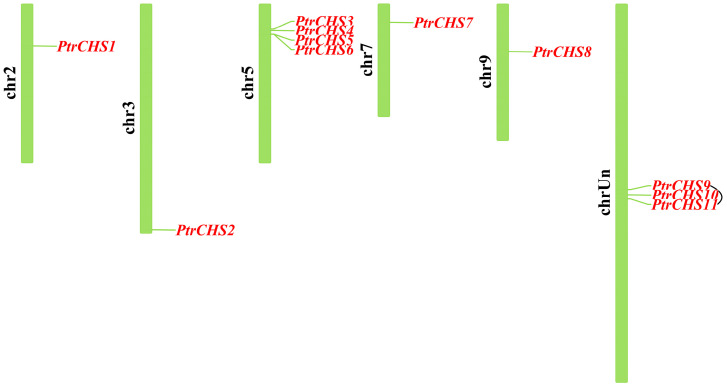
Chromosome localization of the *CHS* family genes in *Poncirus trifoliata*.

A total of 11 members of the *CHS* gene family were identified in the *Poncirus trifoliata* genome using BLAST and HMM technology approaches. These genes were designated as *PtrCHS1* to *PtrCHS11* based on their chromosomal locations (Table 1 and Figure 1). Specifically, *PtrCHS1* is located on chromosome 2; *PtrCHS2* on chromosome 3; *PtrCHS3* to *PtrCHS6* on chromosome 5; *PtrCHS7* on chromosome 7; *PtrCHS8* on chromosome 9; and *PtrCHS9* to *PtrCHS11* are located on unassigned chromosomes. The amino acid lengths of the CHS proteins in *Poncirus trifoliata* range from 195 to 396 residues. Their molecular weights range between 20.88 and 43.50 kDa, and their isoelectric points range from 4.62 to 6.80. Additionally, their instability indices fall within the range of 29.90 to 48.96. The instability index values of PtrCHS2, PtrCHS3, PtrCHS9, and PtrCHS11 are all above 40, suggesting that these are unstable proteins. In silico subcellular localization was predicted using the online tool CELLO v2.5, and the results indicated that all CHS proteins in *Poncirus trifoliata* are localized in the cytosol.

### 2.2. Gene Structure and Protein Conserved Motif Analysis of the CHS Family in Poncirus trifoliata

**Figure 2:**
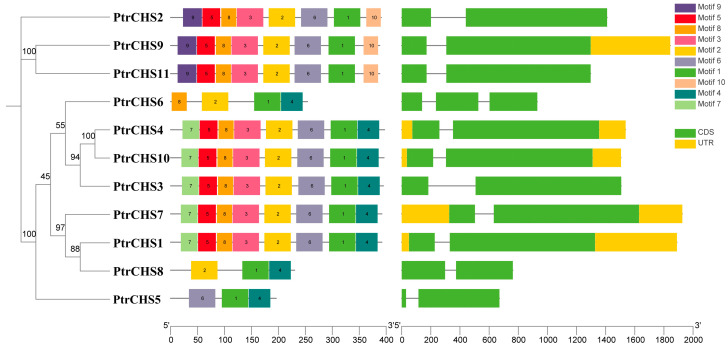
Gene structure and conserved motifs of the *CHS* family members in *Poncirus trifoliata*.

Analyzing the gene structure and conserved motifs among members of a gene family can provide important insights into their functional characteristics. As shown in Figure 2, Except for *PtrCHS6*, all genes contain two exons and one intron. The conserved motif analysis revealed that *Poncirus trifoliata CHS* members contain between 3 and 8 conserved motifs, with considerable variation. However, all *CHS* members possess Motif 1. A detailed analysis showed that *PtrCHS2*, *PtrCHS9*, and *PtrCHS11* share eight motifs (lacking Motif 4), while *PtrCHS1*, *PtrCHS3*, *PtrCHS4*, *PtrCHS7*, and *PtrCHS10* also with eight motifs but lacking Motif 9; *PtrCHS6* contains four motifs; whereas *PtrCHS5* and *PtrCHS8* have only three motifs each.

### 2.3. Phylogenetic Analysis of CHS Family Members in Poncirus trifoliata

**Figure 3:**
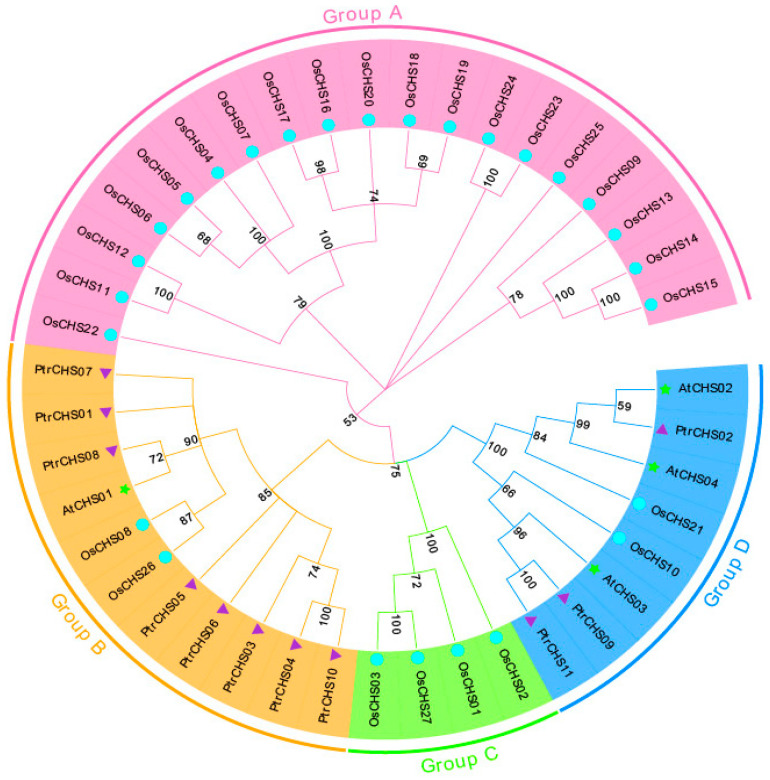
Phylogenetic tree of the *CHS* family proteins from *Poncirus trifoliata*, *Arabidopsis*, and rice. The triangle symbol corresponds to *Poncirus trifoliata*, the circle to rice, and the star to *Arabidopsis*.

To investigate the evolutionary relationships between members of the *CHS* family in *Poncirus trifoliata* and those in other model species, a phylogenetic tree was constructed using MEGA 11 software based on the protein sequences of CHS from *Poncirus trifoliata* (11 members), *Arabidopsis* (4 members), and rice (27 members). As shown in Figure 3, the phylogenetic tree is divided into four subfamilies. PtrCHS2, PtrCHS9, and PtrCHS11 are clustered in subfamily D, while the remaining *CHS* genes from *Poncirus trifoliata* are grouped in subfamily B. Additionally, some CHS proteins from *Poncirus trifoliata*, *Arabidopsis*, and rice are found within the same clade, suggesting that the *CHS* genes existed before the divergence of monocotyledons and dicotyledons.

### 2.4. Secondary and Tertiary Structure Prediction and Phosphorylation Site Analysis of PtrCHS Proteins

The PtrCHSs proteins are primarily composed of α-helices, extended strands, and random coils, with no β-sheets detected. Except for the PtrCHS8 protein, which shows a structural distribution pattern of α-helix = random coil > extended strand, the other ten PtrCHS proteins exhibit a consistent pattern: random coil > α-helix > extended strand. Among them, PtrCHS2 has the highest proportion of random coils at 48.21%, while PtrCHS8 has the lowest at 42.36%. In contrast, PtrCHS8 has the highest proportion of α-helices (42.36%), whereas PtrCHS5 has the lowest (35.38%). Phosphorylation site prediction for PtrCHS protein indicates that serine and threonine residues are the most frequently phosphorylated, while lysine residues are the least common (Table 2).

**Figure 4:**
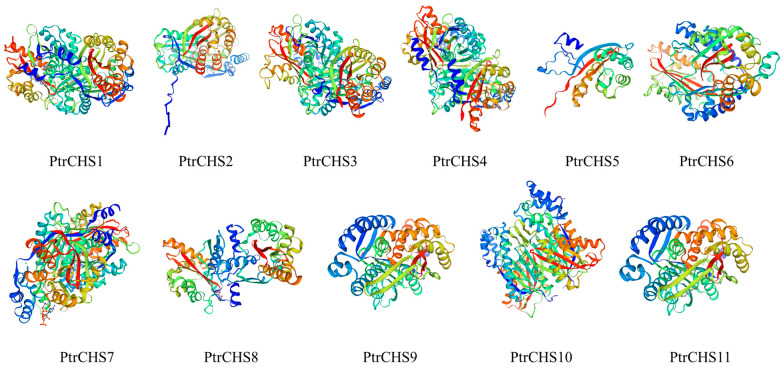
Tertiary structure of the PtrCHS proteins. A blue-to-red color gradient (blue-green-yellow-red) is used to depict the polypeptide chain from the N- to the C-terminus.

Tertiary structure analysis indicates that the protein tertiary structure of PtrCHS9 and PtrCHS11 are highly similar, while the remaining nine proteins show relatively low structural similarity (Figure 4).

### 2.5. Collinearity Analysis of CHS Gene Family in Poncirus trifoliata

To explore the evolutionary relationships among *PtrCHS* family genes, a collinearity analysis was performed. The intra-species analysis identified one collinear gene pair in *Poncirus trifoliata*, specifically between *PtrCHS9* and *PtrCHS11*. 

**Figure 5:**
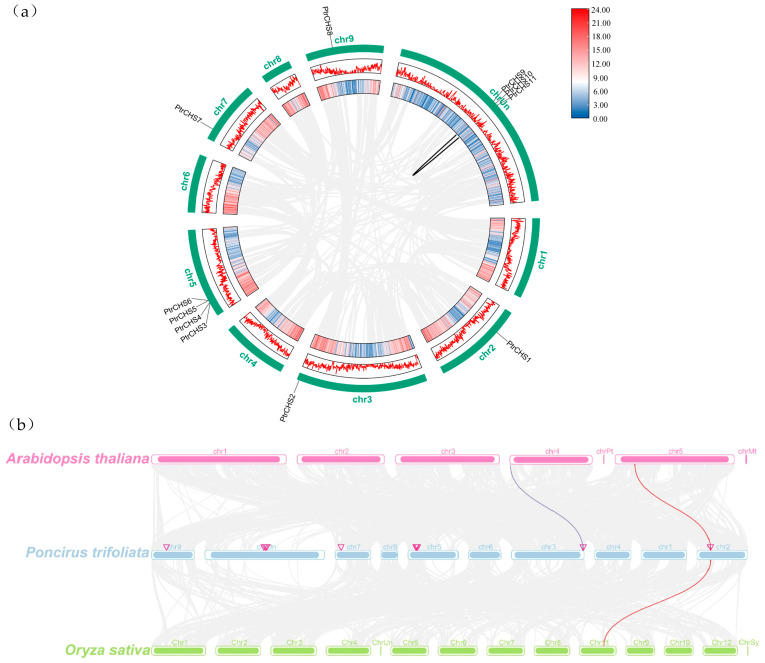
Collinearity analysis of the *CHS* genes. (**a**) Collinearity analysis of the *CHS* genes in *Poncirus trifoliata*. Gray lines represent all syntenic gene pairs in the *Poncirus trifoliata* genome. The solid lines highlight collinear relationships specifically between *PtrCHS* genes. (**b**) Collinearity analysis of the *CHS* genes between *Poncirus trifoliata* and *Arabidopsis*, as well as between *Poncirus trifoliata* and rice. The purple and red solid lines highlight collinear relationships specifically between *CHS* genes.

Inter-species collinearity analysis was then performed to clarify the evolutionary relationships between *PtrCHS* genes and those in *Arabidopsis* and rice. The results revealed two collinear gene pairs between *Poncirus trifoliata* and *Arabidopsis*, and one pair between *Poncirus trifoliata* and rice, suggesting a closer evolutionary relationship with *Arabidopsis* (Figure 5).

### 2.6. Cis-Acting Element Analysis of CHS Genes in Poncirus trifoliata

**Figure 6:**
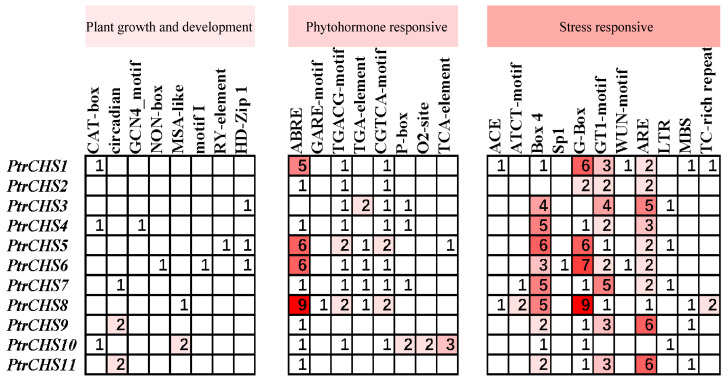
Distribution of cis elements in the promoter sequences of *PtrCHS* genes.

To investigate the regulatory mechanisms of *CHS* genes in *Poncirus trifoliata*, the distribution of cis-acting elements in these genes was analyzed using the PlantCARE online tool. The results showed that the *CHS* family in *Poncirus trifoliata* contains 27 types of cis-acting elements, which can be classified into three major categories: growth and development elements (8 types), hormone response elements (8 types), and stress response elements (11 types) (Figure 6). The growth and development elements mainly include circadian rhythm-responsive elements (circadian, 5), meristem tissue specificity elements (CAT-box, 3), elements involved in palisade mesophyll cell differentiation (HD-Zip 1, 3), and cell cycle regulatory elements (MSA-like, 3). The hormone response elements mainly consist of abscisic acid-responsive elements (ABRE, 32), MeJA-responsive elements (TGACG-motif and CGTCA-motif, 22), auxin-responsive elements (TGA-element, 6), and gibberellin-responsive elements (P-box and GARE-motif, 6). Stress response elements primarily included light-responsive elements (such as Box 4 and G-Box), anaerobic induction elements (ARE, 31), low-temperature-responsive elements (LTR, 4), and drought-inducible elements (MBS, 4). Interestingly, specific responsive elements have been identified in the promoters of a few *CHS* genes in *Poncirus trifoliata*. For example, an endosperm expression regulatory element (GCN4_motif) is present in the promoter of *PtrCHS4*; a seed-specific regulatory element (RY-element) is found in the promoter of *PtrCHS5*; and a root-specific regulatory element (motif I) is detected in the promoter of *PtrCHS6*.

### 2.7. Tissue Expression Pattern Analysis of CHS Genes in Poncirus trifoliata

**Figure 7:**
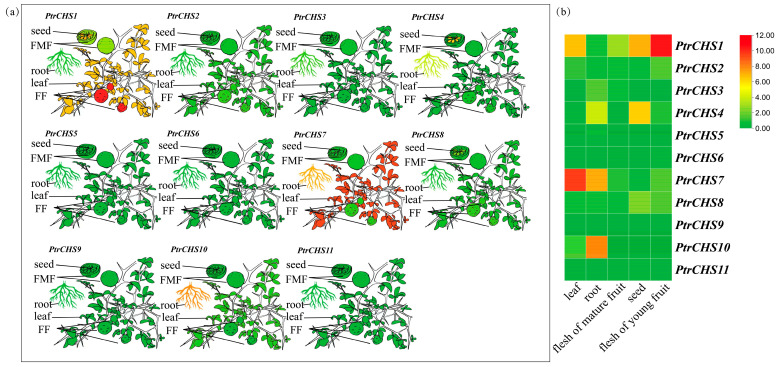
Expression Analysis of the *CHS* genes family members in different tissues of *Poncirus trifoliata*. (**a**) Schematic diagram of expression levels for each gene in different tissues; FF: flesh of young fruit; FMF: flesh of mature fruit; (**b**) heatmap of expression levels. Red color indicates high expression levels, while green denotes low expression levels.

The tissue-specific expression patterns of 11 *CHS* genes in *Poncirus trifoliata* were analyzed using the Citrus Pan-genome Breeding Database (CPBD) (http://citrus.hzau.edu.cn/) citrus (Figure 7 and Table S1). The results indicated distinct expression differences in *PtrCHS* genes across seeds, roots, leaves, flesh of young fruit, and flesh of mature fruit. Most *PtrCHS* genes exhibited low expression levels, with only *PtrCHS1* showing relatively high expression in seeds, leaves, and flesh of young fruit. Among all *PtrCHS* genes, *PtrCHS7* was highly expressed in leaves, while *PtrCHS10* showed the highest expression in roots. In the remaining tissues, *PtrCHS1* had the highest expression levels. In contrast, *PtrCHS5*, *PtrCHS6*, *PtrCHS9*, and *PtrCHS11* consistently showed low expression across all five tissues analyzed. Notably, *PtrCHS5* was detected at low levels only in roots, and *PtrCHS9* was not detected in any of the five tissues. In addition, several *PtrCHS* genes (*PtrCHS3*, *PtrCHS4*, *PtrCHS7*, and *PtrCHS10*) were found to be highly expressed in roots.

### 2.8. Expression Pattern Analysis of PtrCHS Under Salt Treatment

**Figure 8:**
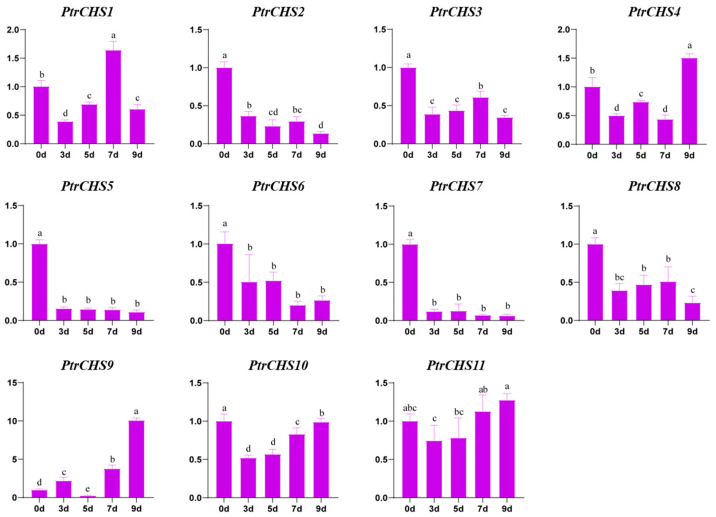
Expression Analysis of *PtrCHSs* in salt stress. Different letters indicate significant differences (ANOVA analysis, *p* < 0.05).

To investigate the role of *PtrCHS* genes in response to salt stress, 11 *PtrCHS* genes were treated with 200 mM NaCl, and their expression patterns were analyzed using the RT-qPCR method, as shown in Figure 8. Except for *PtrCHS1*, *PtrCHS4*, *PtrCHS9*, and *PtrCHS11*, the expression of the remaining genes was inhibited under salt stress. Among these four genes, *PtrCHS11* showed no significant change in expression. The expression level of *PtrCHS1* followed a trend of decrease–increase–decrease, and it reached the highest level on the 7th day of salt treatment, which was higher than that of the untreated samples. The expression of *PtrCHS4* displayed a fluctuating pattern of decrease–increase– decrease–increase, with a significantly higher level observed on day 9 compared to other time points. Notably, the overall expression level of *PtrCHS9* was significantly upregulated-except at 7 d-reaching its peak at 9 d, which was 10.09 times higher than that of the control and surpassed the expression levels of other *CHS* genes. These results indicate that, except for *PtrCHS11*, all other *CHS* genes can respond to salt stress and exhibit a dynamic and member-specific response patterns.

### 2.9. Expression Pattern Analysis of CHS Genes in Poncirus trifoliata Under Cold Treatment

**Figure 9:**
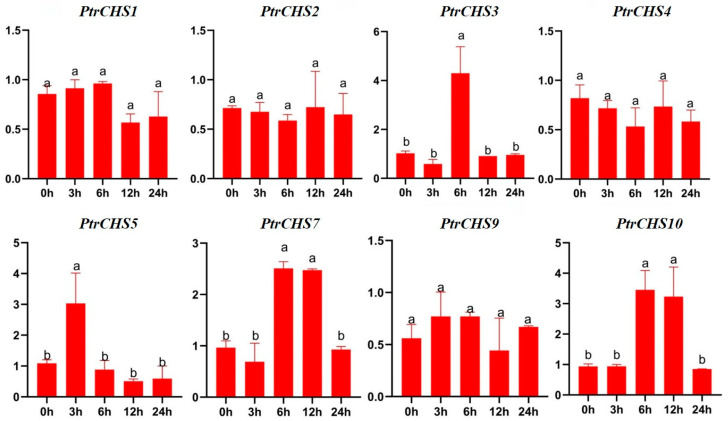
Expression Analysis of *PtrCHSs* in low temperature stress. Different letters indicate significant differences (ANOVA analysis, *p* < 0.05).

Cis-element analysis revealed that four *CHS* genes in *Poncirus trifoliata* contain low-temperature-responsive elements. Therefore, the RT-qPCR method was used to analyze the expression of four *CHS* genes with low-temperature-responsive elements (*PtrCHS3*, *PtrCHS5*, *PtrCHS7*, and *PtrCHS10*) and four *CHS* genes lacking such elements (*PtrCHS1*, *PtrCHS2*, *PtrCHS4*, and *PtrCHS9*) under cold treatment (Figure 9). The results showed that the four *CHS* genes without low-temperature-responsive elements did not exhibit significant changes in expression within 24 h of cold treatment. In contrast, the four *PtrCHS* genes containing low-temperature-responsive elements were significantly upregulated at different time points, mainly at 3 h (*PtrCHS5*), 6 h (*PtrCHS3*, *PtrCHS7*, and *PtrCHS10*), and 12 h (*PtrCHS7* and *PtrCHS10*). Both *PtrCHS7* and *PtrCHS10* were significantly upregulated at both time points. These results suggest that *PtrCHS7* and *PtrCHS10* may be key genes involved in the response to cold stress, potentially playing important roles in the regulation of cold tolerance in *Poncirus trifoliata*. Therefore, *PtrCHS7* and *PtrCHS10* were cloning from *Poncirus trifoliata*, and their overexpression vectors were constructed for subsequent functional analysis (Figures S1–S5 and Table S2).

## 3. Discussion

Due to its strong cold resistance and good graft compatibility with most cultivated citrus varieties, *Poncirus trifoliata* has been widely used as an important rootstock in the citrus industry [40,41]. CHS plays a crucial role in plant growth and development as well as in responses to environmental (or abiotic) stress [42,43]. However, no comprehensive analysis has been specifically conducted on the *CHS* genes family in *Poncirus trifoliata* (*PtrCHSs*). In this study, 11 *CHS* genes were identified from the *Poncirus trifoliata* genome using bioinformatics approaches (Table 1). Compared with the number of *CHS* genes reported in other plant species (ranging from 7 to 27), the *CHS* gene family in *Poncirus trifoliata* is of medium size.

The *CHS* genes in *Poncirus trifoliata* are primarily located on five known chromosomes (Figure 1), with each gene containing 2 to 3 exons (Figure 2). The number of *CHS* gene exons in other plant species is also predominantly between 2 and 3, indicating that the *CHS* gene structure is relatively conserved [7,8,9]. All CHS proteins in *Poncirus trifoliata* are predicted to be localized in the cytoplasm (Table 1), and similar patterns have been observed in other plant species. For example, among the 27 CHS proteins in rice, 21 are predicted to be localized in the cytosol [6]; among the 16 CHS proteins in chrysanthemum, 10 are predicted to be localized in the cytosol [10]; all 14 CHS proteins in soybean are localized in the cytosol, with 5 of them also localized in the nucleus [8]. These localization results suggest that CHS primarily functions in the cytosol.

Phylogenetic analysis classified the *CHS* family members into four subgroups (Figure 3). However, *CHS* family members from *Poncirus trifoliata* were clustered only within the Group B and Group D clades. This result indicated that some *CHS* genes from *Poncirus trifoliata* existed before the divergence of monocotyledons and dicotyledons, as they clustered within the same clade as *Arabidopsis* and rice. This suggests that the *CHS* function has been prioritized during plant evolution. Supporting this notion, previous studies have found that the *CoCHS* in *Coelogyne ovalis* Lindl. is present in monocotyledonous plant groups, further suggesting that the *CHS* genes existed prior to the divergence of monocotyledons and dicotyledons [44]. PtrCHS9 and PtrCHS11 share a common tertiary structure. The structural differences observed in PtrCHS2, a member belonging to the same motif group as PtrCHS9/11, suggest that subtle sequence variations outside the core motif can also significantly influence protein folding. In contrast, the simplified structures of PtrCHS5, PtrCHS6, and PtrCHS8 may stem from their sparse motifs. This study identified a total of four collinear relationships through collinearity analysis of *CHS* genes in three different species, including two between *Poncirus trifoliata* and *Arabidopsis*, and one with rice (Figure 5). Similarly, four collinearity relationships have been identified for *Phaseolus vulgaris CHS3*, one of which is related to *Arabidopsis* [11].

The distribution of cis-acting elements in the promoter region of the *Poncirus trifoliata CHS* genes indicates that the *CHS* family is enriched with hormone response element and stress response element (Figure 6). Among these, abscisic acid response elements, MeJA response elements, light-responsive elements, and anaerobic induction elements are particularly abundant. The distribution pattern of cis-acting elements in the *CHS* promoter of *Poncirus trifoliata* is consistent with previous research findings [6,9,10].

*MaCHS4* in mulberry is highly expressed in roots and leaves, while *MaCHS1* and *MaCHS2* are predominantly expressed in leaves [19]. *SmCHS7* and *SmCHS8* are mainly expressed in the roots and stems of *S. miltiorrhiza*, whereas *SmCHS2* and *SmCHS6* show the highest expression levels in flowers [45]. In peas, *CHS1* and *CHS3* are expressed in petals and roots, while *CHS2* expression is detected only in roots [46]. These findings indicate that *CHS* exhibits tissue specificity in most plants. In this study, *PtrCHS7* and *PtrCHS10* in *Poncirus trifoliata* were found to have the highest expression levels in leaves and roots, respectively, while *PtrCHS1* showed the highest expression in flesh of young fruits (Figure 7). This indicates that *CHS* genes in *Poncirus trifoliata* also exhibit tissue specificity, and different *CHS* genes may serve distinct physiological functions in the plant.

*MaCHS1* in mulberry is rapidly expressed under salt treatment, reaching a peak at 24 h [19]. In this study, the expression of *PtrCHS* under salt stress was analyzed to explore its response to salt stress. Interestingly, it was found that *PtrCHS9* showed significant upregulation after 9 days of salt treatment (Figure 8). In contrast, *PtrCHS1*, which exhibits constitutive expression across various tissues (Figure 7), displayed a more dynamic and moderate fluctuation in expression under salt stress. Therefore, we hypothesize that *PtrCHS9* might be a key gene, possibly playing a role in the citrus salt stress response mechanism by scavenging ROS or through other pathways [47], while *PtrCHS1* could serve as a foundational component of the stress tolerance background. However, the specific functional mechanisms and potential synergy between constitutive and inducible *CHS* members still require further experimental verification.

Studies have shown that low-temperature-responsive elements have been identified in the *CHS* genes promoter regions of plants such as *Chrysanthemum nankingense*, *Zanthoxylum bungeanum*, and *Dendrobium catenatum* [10,21,48]. In addition, significant upregulation of certain *CHS* genes in *Dendrobium catenatum* has been observed under cold stress [48]. In the present study, four *CHS* genes of *Poncirus trifoliata* within the *CHS* gene family—*PtrCHS3*, *PtrCHS5*, *PtrCHS7*, and *PtrCHS10*—were found to contain low-temperature-responsive elements in their promoters (Figure 6). Therefore, this study focused on analyzing the expression patterns under cold stress of these four *CHS* genes with low-temperature-responsive elements (*PtrCHS3*, *PtrCHS5*, *PtrCHS7,* and *PtrCHS10*) as well as four *CHS* genes lacking such elements (*PtrCHS1*, *PtrCHS2*, *PtrCHS4*, and *PtrCHS9*). As expected, the four *CHS* genes containing low-temperature-responsive elements were significantly upregulated at various time points, with *PtrCHS7* and *PtrCHS10* showing sustained and marked upregulation at both the 6 h and 12 h time points (Figure 9). Therefore, *PtrCHS7* and *PtrCHS10* are likely to play important regulatory roles in cold stress. These two candidate genes should be prioritized in subsequent studies on biological function. Furthermore, the upregulation of *PtrCHS7* and *PtrCHS10* under cold stress, along with *PtrCHS9* under salt stress, is predicted to enhance the biosynthesis of chalcone and subsequent flavonoid derivatives. These compounds act as critical secondary metabolites that serve antioxidant functions, which efficiently scavenging excess ROS to protect cellular structures from oxidative damage and stabilize membrane integrity under stress conditions [49]. Thus, the induced expression of these *PtrCHS* genes is likely implicated in enhancing cellular antioxidant capacity and fortifying the physiological defense mechanisms of *Poncirus trifoliata* against environmental adversities.

Currently, our research group has successfully completed the cloning of *PtrCHS7* and *PtrCHS10* in *Poncirus trifoliata*, and has constructed their overexpression vectors. The next step will involve constructing gene editing vectors, followed by stable genetic transformation to obtain transgenic materials, to further validate their cold resistance function and investigate the underlying molecular mechanisms.

In this study, 11 *CHS* gene members were identified in *Poncirus trifoliata* using bioinformatics approaches. Their physicochemical properties, gene structures, promoter cis-acting elements, and tissue-specific expression profiles were analyzed. Gene expression patterns under salt stress and cold stress were also examined, leading to the identification of two key *CHS* genes responsive to low temperature (*PtrCHS7* and *PtrCHS10*). These findings provide a theoretical foundation and reference for citrus cold resistance molecular breeding.

## 4. Materials and Methods

### 4.1. Identification of Chalcone Synthase Gene Family Members in Poncirus trifoliata

The complete genome, protein sequences, and annotation information of *Poncirus trifoliata* were downloaded from CPBD (http://citrus.hzau.edu.cn/index.php, accessed on 11 October 2024). Relevant protein sequences for *Arabidopsis* were obtained from Ensembl Plants (http://plants.ensembl.org/, accessed on 11 October 2024). All CHS protein sequences from *Arabidopsis* were used as queries to perform a BLAST search against the *Poncirus trifoliata* protein database using TBtools v2.332, with the E-value threshold set to e^−5^. To enhance the reliability of candidate gene identification, a Hidden Markov Model (HMM) search was also performed using HMMER v3.3.2 with the canonical CHS domain profiles (PF00195 and PF02797) obtained from the Pfam database (http://pfam.xfam.org/, accessed on 11 October 2024). The results from both the BLAST and HMM searches were combined to compile a comprehensive list of initial candidates. Candidate CHS protein sequences in *Poncirus trifoliata* were identified and subsequently validated for the presence of conserved protein domains using the NCBI Conserved Domain Search Service (https://www.ncbi.nlm.nih.gov/Structure/cdd/wrpsb.cgi, accessed on 11 October 2024). Only hits containing both the Chal_sti_synt_N and Chal_sti_synt_C domains were designated as bona fide *CHS* members.

### 4.2. Bioinformatics Analysis of the Chalcone Synthase Gene Family in Poncirus trifoliata

The physicochemical properties of the *CHS* family members in *Poncirus trifoliata* were predicted using TBtools v2.332. In silico subcellular localization of the CHS protein family in *Poncirus trifoliata* was predicted using the online tool CELLOv.2.5 (https://cello.life.nctu.edu.tw/, accessed on 11 October 2024). The protein sequences of all identified *PtrCHS* members were used for conserved motif analysis using the online tool MEME (https://meme-suite.org/meme/tools/meme, accessed on 11 October 2024), with the number of motifs set to 10 and all other parameters kept at default settings. Gene structure and motif visualization were performed using TBtools v2.332. Multiple sequence alignment was conducted in MEGA 11 using the amino acid sequences of CHS protein from *Poncirus trifoliata*, *Arabidopsis*, and rice. The CHS-related protein sequence data for rice were also obtained from Ensembl Plants (http://plants.ensembl.org/, accessed on 11 October 2024). A phylogenetic tree was constructed using the maximum likelihood method based on the Jones-Taylor-Thornton (JTT) model, with 1000 bootstrap replicates and all other parameters kept at their default settings. The phylogenetic tree was edited and refined using Evolview (http://www.evolgenius.info/evolview/#/treeview, accessed on 11 October 2024). The secondary and tertiary structures of the proteins were predicted using SOPMA (https://npsa.lyon.inserm.fr/, accessed on 10 June 2025) and SWISS-MODEL (https://swissmodel.expasy.org/, accessed on 10 June 2025) online platform, respectively. Phosphorylation sites were predicted using CBS-NetPhos 3.1 (https://services.healthtech.dtu.dk/services/NetPhos-3.1/, accessed on 10 June 2025) with a prediction threshold of 0.5. The MCScanX tool in TBtools v2.332 was used to analyze collinearity within the *Poncirus trifoliata* genome, as well as between *Poncirus trifoliata* and the model species *Arabidopsis* and rice.

A 2000 bp sequence upstream of the transcription start site of each *CHS* gene in *Poncirus trifoliata* was selected as the promoter region. All cis-acting elements within these promoter regions were identified using the online tool PlantCARE (http://bioinformatics.psb.ugent.be/webtools/plantcare/html/, accessed on 11 October 2024). Key responsive elements (such as hormone- and stress-related elements) were then categorized based on their biological functions, and their distribution and frequency across different *PtrCHS* promoters were statistically analyzed and compared. RNA-seq expression data for the *CHS* gene family in *Poncirus trifoliata* were obtained from the CPBD, and gene expression heatmaps were generated using TBtools software.

### 4.3. Expression Analysis of PtrCHSs Under Salt and Cold Stress

*Poncirus trifoliata* plants were provided by the Citrus Research Team at the College of Horticulture and Landscape Architecture, Yangzhou University. All plants used were 45-day-old seedlings. The plants were cultivated under controlled conditions at 25 °C with 60% humidity, a 16 h light period, and an 8 h dark period. Uniformly growing seedlings were selected and treated with 200 mM NaCl. Treatment durations were set at 0, 3, 5, 7, and 9 days, with seedlings irrigated with distilled water serving as the control group. For cold treatment, uniformly grown seedlings were exposed to 4 °C, and leaf samples were collected at 0, 3, 6, 12, and 24 h, using seedlings maintained at room temperature as the control.

All collected samples were ground in liquid nitrogen, and approximately 1 g was aliquoted and immediately stored at −80 °C. Three biological replicates and three technical replicates were conducted at each time point.

Total RNA was extracted using the CTAB method. RNA quality was evaluated by gel electrophoresis and a bioanalyzer. First-strand cDNA synthesis was carried out using the HiScript First Strand cDNA Synthesis Kit (Vazyme, Nanjing, China). RT-qPCR was performed on the CFX96 Real-Time PCR System (Bio-Rad, Hercules, CA, USA). The β-actin gene of citrus was used as the internal control. The relative transcriptional expression level of the *CHS* gene was calculated using the 2^−∆∆CT^ method. Primers were designed using Primer Premier 5 software, with detailed information provided in Supplementary Table S3. Graphs were generated using GraphPad Prism 9.

### 4.4. Cloning of PtrCHS7 and PtrCHS10 Genes and Overexpression Vectors Construction

Total RNA was extracted using the Universal Plant Total RNA Rapid Extraction Kit (Vazyme, Nanjing, China), followed by reverse transcription to synthesize cDNA. Full-length (FL) coding sequences (CDS) of *PtrCHS7* and *PtrCHS10* were amplified using primers designed with Primer Premier 5 for cloning. The FL sequences of *PtrCHS7* and *PtrCHS10* were 1188 bp and 1176 bp, respectively.

The purified PCR products were recombined with the pDONR222 vector using BP enzyme (Thermo Fisher Scientific, Waltham, MA, USA). The resulting recombinant plasmids, pDONR222-*PtrCHS7* and pDONR222-*PtrCHS10*, were further recombined with the pKGMYC vector and pGWB411 vector, respectively, using LR enzyme (Thermo Fisher Scientific, Waltham, MA, USA) to construct the overexpression vectors (pKGMYC-*PtrCHS7* and pGWB411-*PtrCHS10*).

## 5. Conclusions

In this study, eleven members of the *CHS* gene family (*PtrCHS1*-*11*) were identified in *Poncirus trifoliata* using bioinformatics approaches. The results showed that the *CHS* genes in *Poncirus trifoliata* are distributed across five known and several unknown chromosomes, and all are predicted to be localized in the cytosol.

Gene structure analysis revealed that, except for *PtrCHS6*, all other members contain two exons and one intron. Conserved motif analysis indicated that Motif 1 is conserved across all members. The protein tertiary structures of *PtrCHS9* and *PtrCHS11* were found to be highly similar. Based on phylogenetic analysis, the *CHS* genes of *Poncirus trifoliata* were classified into four subgroups, with some members clustering in the same clade as *CHS* genes from *Arabidopsis* and rice, suggesting that *CHS* genes originated before the divergence of monocotyledonous and dicotyledonous plants. Synteny analysis revealed the presence of one set of collinear gene pairs within *Poncirus trifoliata*, while two sets and one set of collinear gene pairs were identified between *Poncirus trifoliata* and *Arabidopsis*, and between *Poncirus trifoliata* and rice, respectively.

Promoter cis-element analysis revealed that *CHS* genes in *Poncirus trifoliata* contain various hormone response elements and stress response elements. Tissue expression profiling showed that *PtrCHS1* is highly expressed across multiple tissues, while *PtrCHS7* and *PtrCHS10* exhibit tissue-specific high expression in leaves and roots, respectively. Under salt stress, the expression of *PtrCHS9* was significantly upregulated. Analysis under cold stress revealed that *PtrCHS3*, *PtrCHS5*, *PtrCHS7*, and *PtrCHS10*, which contain low-temperature response elements, were significantly upregulation in expression. Among them, *PtrCHS7* and *PtrCHS10* exhibited sustained upregulation at multiple time points, suggesting that they may function as key genes in the cold stress response of *Poncirus trifoliata*.

## Figures and Tables

**Table 1:** Characteristic features of the *CHS* gene family members in *Poncirus trifoliata*.

Gene Name	CPBD Number	Chromosome	Number of Amino Acids (aa)	Molecular Weight/kDa	Isoelectric Point	The Instability Index	In Silico Subcellular Localization Predicted
*PtrCHS1*	Pt2g009170.1	Chr2	391	42.62	6.29	35.00	Cytosol
*PtrCHS2*	Pt3g040440.1	Chr3	390	42.89	5.81	48.96	Cytosol
*PtrCHS3*	Pt5g007170.1	Chr5	394	43.42	6.80	42.09	Cytosol
*PtrCHS4*	Pt5g007600.1	Chr5	396	43.35	6.33	37.25	Cytosol
*PtrCHS5*	Pt5g008530.1	Chr5	195	20.88	4.62	29.90	Cytosol
*PtrCHS6*	Pt5g008550.1	Chr5	253	27.62	5.90	36.37	Cytosol
*PtrCHS7*	Pt7g002450.1	Chr7	391	42.76	5.61	38.31	Cytosol
*PtrCHS8*	Pt9g010550.1	Chr9	229	24.69	5.57	38.80	Cytosol
*PtrCHS9*	PtUn017340.1	ChrUn	387	42.44	5.87	43.29	Cytosol
*PtrCHS10*	PtUn017780.1	ChrUn	395	43.50	6.17	32.10	Cytosol
*PtrCHS11*	PtUn018300.1	ChrUn	387	42.42	5.87	43.29	Cytosol

**Table 2:** Structural characteristics of PtrCHS proteins.

Protein Name	Secondary Structure				Phosphorylation Amino Acid Number		
	Alpha Helix (%)	Extended Strand (%)	Beta Turn (%)	Random Coil (%)	Serine	Threonine	Tyrosine
PtrCHS1	38.87	14.83	0.00	46.29	16	13	5
PtrCHS2	38.21	13.59	0.00	48.21	17	14	3
PtrCHS3	40.61	14.47	0.00	44.92	17	13	6
PtrCHS4	38.89	14.65	0.00	46.46	19	9	5
PtrCHS5	35.38	16.92	0.00	47.69	9	6	1
PtrCHS6	36.36	17.39	0.00	46.25	11	6	1
PtrCHS7	40.41	15.09	0.00	44.50	14	12	3
PtrCHS8	42.36	15.28	0.00	42.36	8	4	0
PtrCHS9	39.79	14.21	0.00	45.99	16	12	5
PtrCHS10	39.49	14.68	0.00	45.82	14	7	5
PtrCHS11	40.83	15.5	0.00	43.67	15	12	5

## Data Availability

All data, tables, and figures in this manuscript are original and data are contained within the article and the Supplementary Materials.

## Supplementary Materials

The following supporting information can be downloaded at: https://www.mdpi.com/article/10.3390/plants14193003/s1, Figure S1. The electrophoretic result after PCR amplification of *PtrCHS7* and *PtrCHS10*; Figure S2. The sequence alignment for the cloning of *PtrCHS7*; Figure S3. The sequence alignment for the cloning of *PtrCHS10*; Figure S4. The sequencing result of the pKGMYC*-PtrCHS7* overexpression vector; Figure S5. The sequencing result of the pGWB411*-PtrCHS10* overexpression vector; Table S1. The expression levels of *PtrCHSs* in various tissues of *Poncirus trifoliata*; Table S2. Primers used for cloning genes and overexpression vectors construction; Table S3. Primers used for RT-qPCR of *PtrCHSs*.
