# Supplementary material for: Identification of Chalcone Synthase Genes and Their Responses to Salt and Cold Stress in Poncirus trifoliata
Source: Plants (Basel). 2025 Sep 28;14(19):3003. doi: 10.3390/plants14193003 (PMC12525817; doi:10.3390/plants14193003)
Supplement: Supplementary file 1 [file plants-14-03003-s001.zip › Supplementary figure S1-5.pdf]

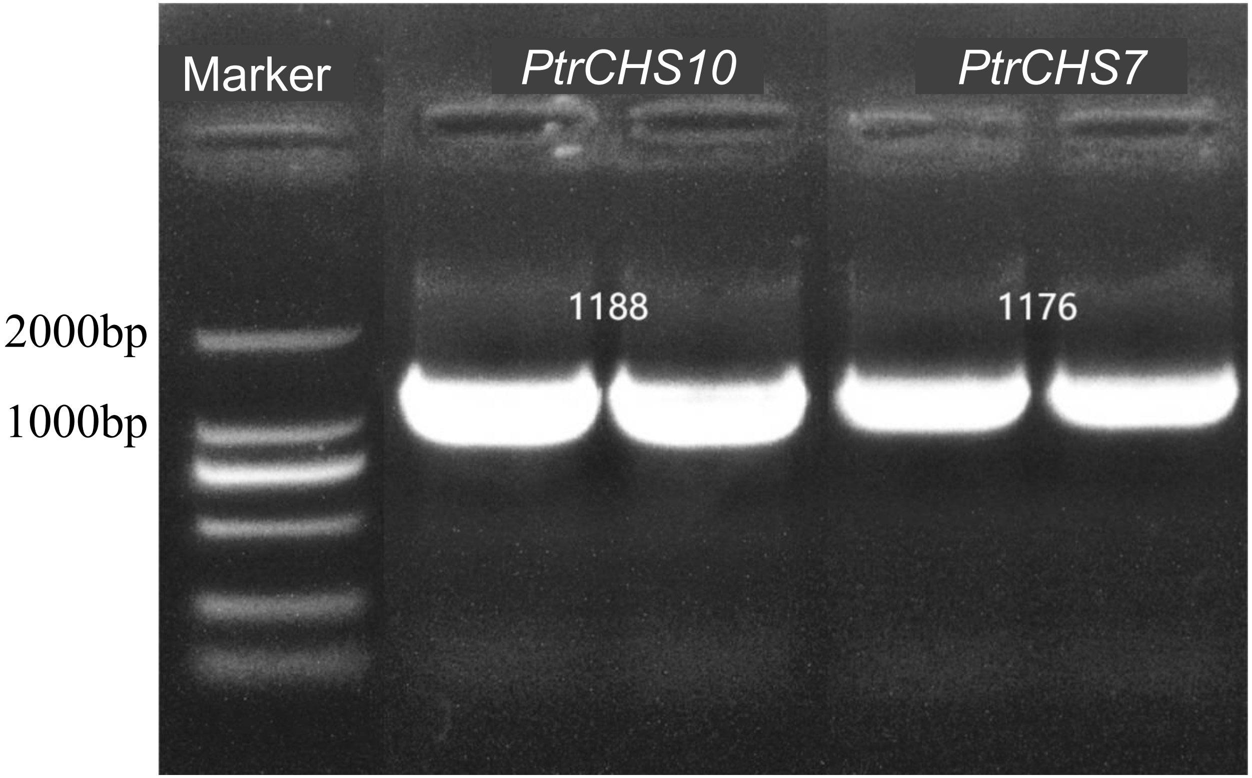

Figure S1. The electrophoretic result after PCR amplification of *PtrCHS7* and *PtrCHS10*.

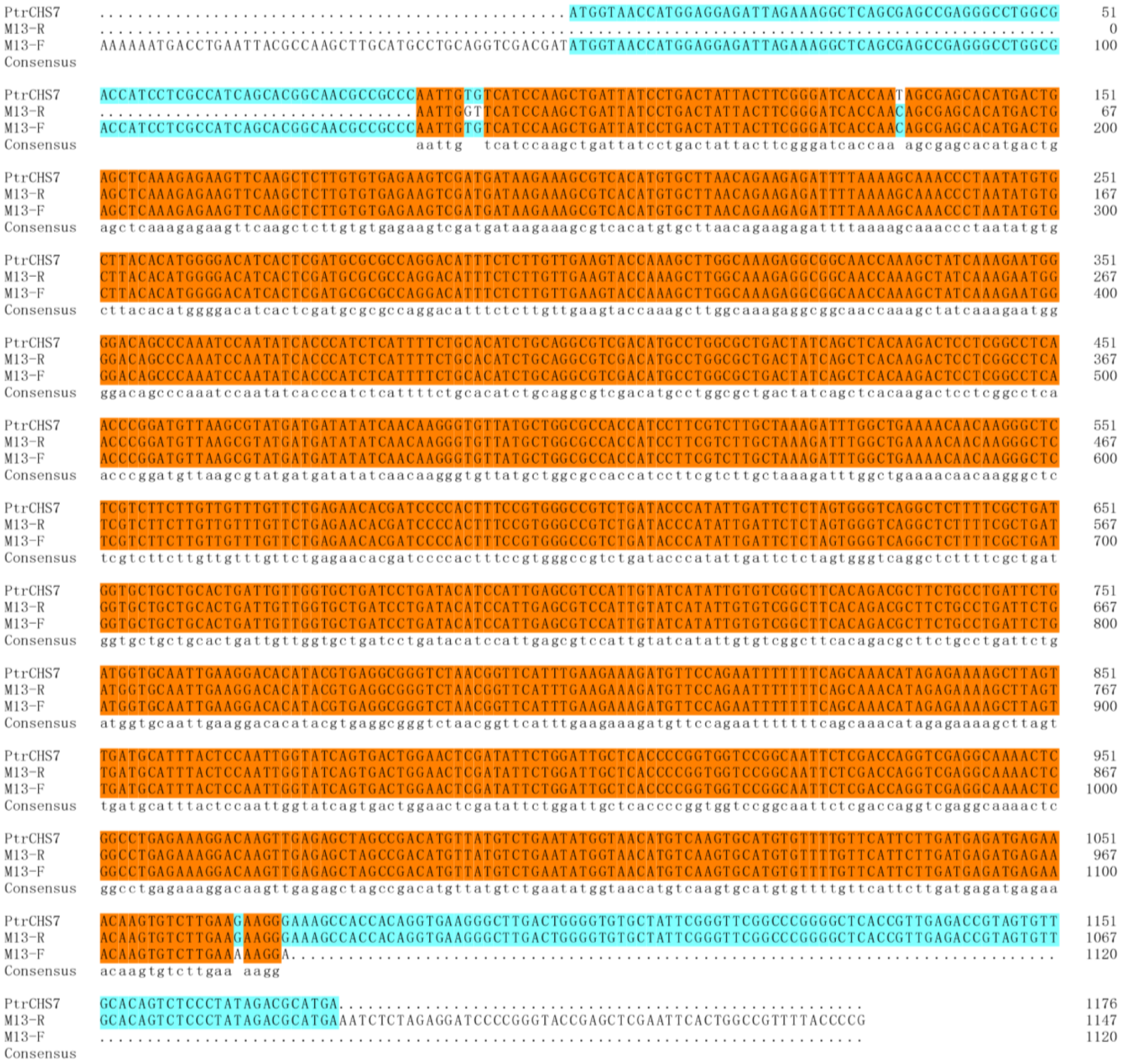

Figure S2. The sequence alignment for the cloning of *PtrCHS7*.

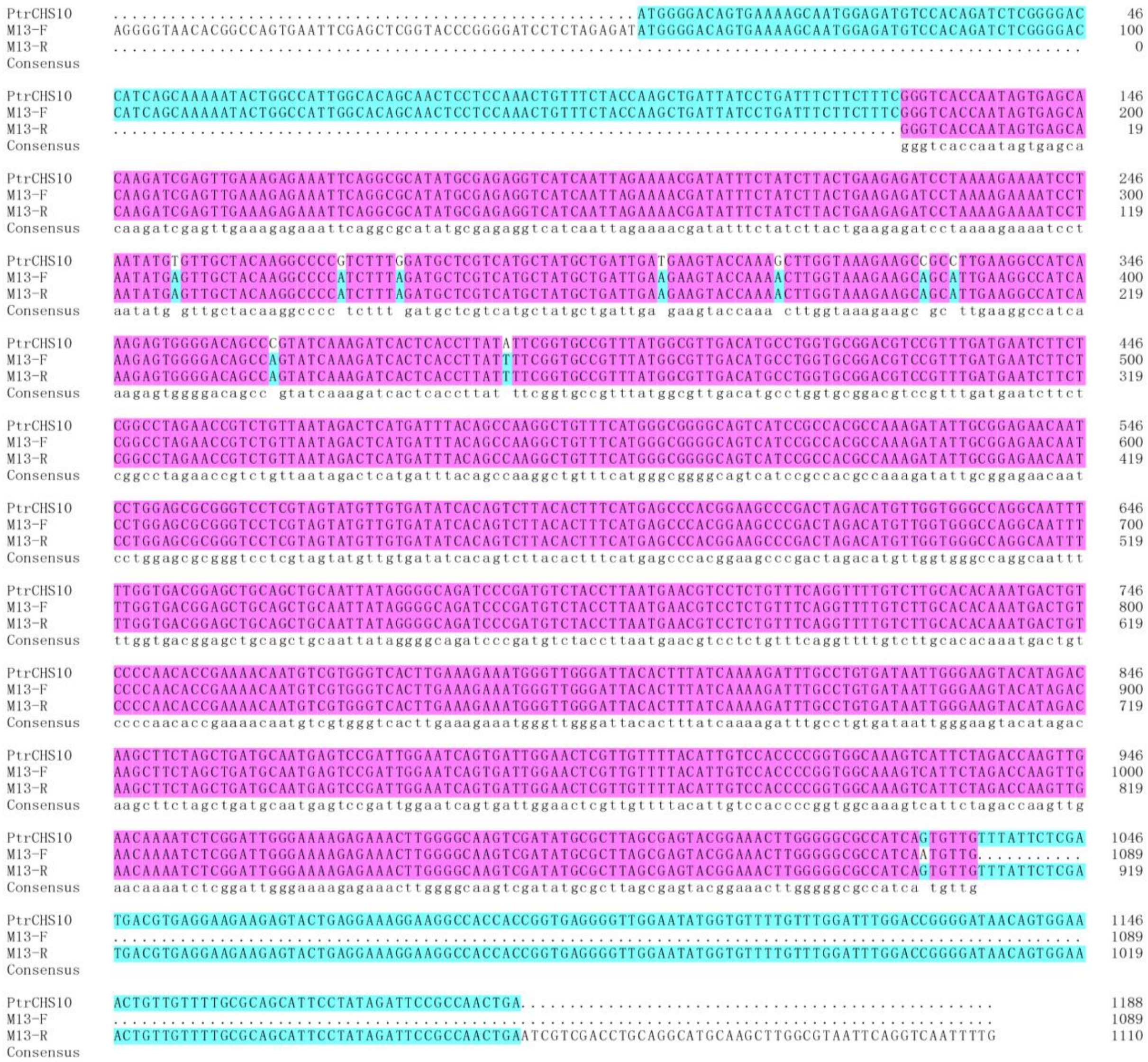

Figure S3. The sequence alignment for the cloning of *PtrCHS10*.

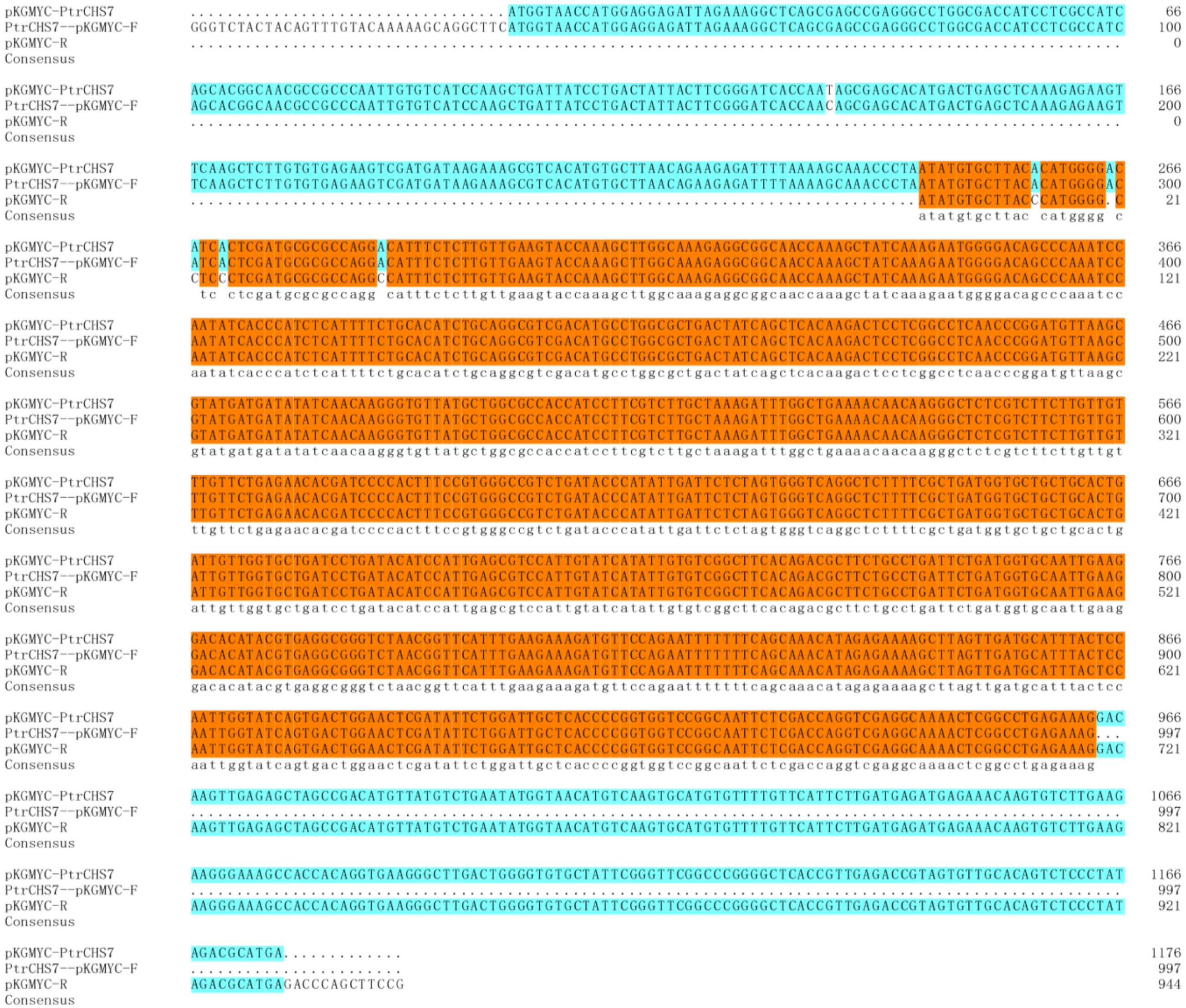

Figure S4. The sequencing result of the pKGMYC-*PtrCHS7* overexpression vector.

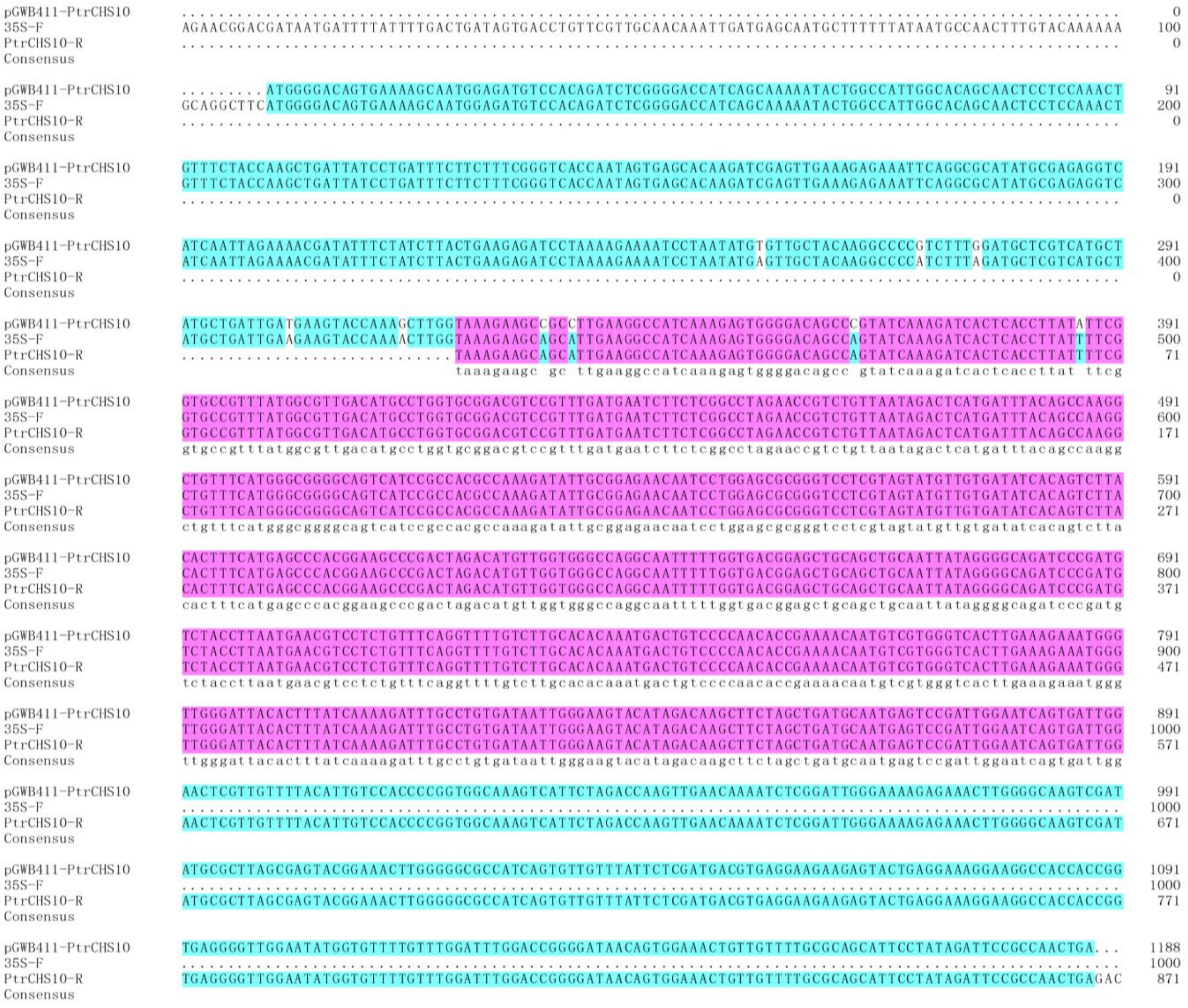

Figure S5. The sequencing result of the pGWB411-*PtrCHS10* overexpression vector.
